# A Novel Human Stem Cell Culture Model for Severe Traumatic Brain Injury Reflecting Sexual Dimorphism in Heterotopic Ossification

**DOI:** 10.3390/cells14191491

**Published:** 2025-09-24

**Authors:** Jonas Joneleit, Philipp Leimkühler, Tarek Niemann, Matthias Ruwe, Christian Jantos, Dirk Wähnert, Christian Kaltschmidt, Thomas Vordemvenne, Barbara Kaltschmidt

**Affiliations:** 1 Molecular Neurobiology, Bielefeld University, Universitätsstrasse 25, 33615 Bielefeld, Germanybarbara.kaltschmidt@uni-bielefeld.de (B.K.); 2 Forschungsverbund BioMedizin Bielefeld/OWL FBMB e.V., Maraweg 21, 33617 Bielefeld, Germany; christian.jantos@evkb.de (C.J.); dirk.waehnert@evkb.de (D.W.); c.kaltschmidt@uni-bielefeld.de (C.K.); thomas.vordemvenne@evkb.de (T.V.); 3 Department of Cell Biology, Bielefeld University, Universitätsstrasse 25, 33615 Bielefeld, Germany; 4 Department of Trauma and Orthopedic Surgery, Protestant Hospital of Bethel Foundation, Campus Bielefeld-Bethel, University Hospital OWL of Bielefeld University, Burgsteig 13, 33617 Bielefeld, Germany; philipp-julian.leimkuehler@evkb.de; 5 Institute of Laboratory Medicine and Microbiology, Protestant Hospital of Bethel Foundation, Campus Bielefeld-Bethel, University Hospital OWL of Bielefeld University, Burgsteig 13, 33617 Bielefeld, Germany

**Keywords:** traumatic brain injury, heterotopic ossification, S100B, TGF-β1, sexual dimorphism, adipose-derived stem cells, skeletal stem cells, osteogenic differentiation

## Abstract

Heterotopic ossification (HO) is a disease characterized by ectopic bone formation, which can occur following severe traumatic brain injury (TBI). However, the underlying mechanisms remain poorly understood. In this study, we established a stem cell model using adipose-derived stem cells (ADSCs) and skeletal stem cells (SSCs) to examine osteogenic factors present in the sera of TBI patients. Incubation of ADSCs and SSCs with osteoinductive medium supplemented with TBI serum significantly enhanced osteogenic differentiation, particularly in male ADSCs and both female and male SSCs, with male SSCs exhibiting the highest osteogenic potential. Furthermore, we identified TGF-β1 as an important factor involved in these osteogenic processes. Elevated levels of TGF-β1 were detected in the serum of male TBI patients 14 days post-injury. Cellular assays revealed a sexual dimorphism in response to TGF-β1 neutralization: osteogenic differentiation in male SSCs was significantly reduced, while no effect was detectable in female SSCs. These findings, together with the rarity of HO in female patients, suggest that TGF-β1 plays a central role in the development of HO in males. Furthermore, this study highlights the importance of considering sex-specific mechanisms in traumatic HO for the development of sex-specific therapy options.

## 1. Introduction

Traumatic brain injury (TBI) is a significant medical condition caused by external mechanical forces impacting the head. It can lead to temporary or permanent impairment of brain function and, in some cases, to abnormal bone formation (heterotopic ossification, HO) [1]. The most common causes of TBI include direct striking of the skull, rapid acceleration/deceleration movements and penetrating injuries [2]. TBI can be classified in a clinical context using the Glasgow Coma Scale (GCS). Through the GCS, different aspects of behavior, including eye opening, motor and verbal responses, are evaluated and assigned a score. Scores range from 3, indicating deep unconsciousness, to 15, which suggests normal neurological conditions [3]. More precisely, a GCS score of 13–15 indicates mild TBI, 9–12 marks moderate TBI and a score of 8 or less reflects severe TBI [4]. Globally, 20,837,465 (95% confidence interval (CI): 18,128,306–23,839,393) new cases of TBI were reported for 2021, with an age-standardized incidence of 259 (CI: 226–296) cases per 100,000 population. Moreover, it was revealed that the global incidence across all age groups was higher in males than in females [5]. An epidemiological study conducted by Dewan and colleagues revealed that 81% of cases are classified as mild, 11% as moderate and 8% as severe [6]. Pathophysiologically, TBI can lead to primary and secondary injuries. Primary injuries manifest as contusions, hemorrhages (epidural, subdural and intracerebral), disruption of the blood–brain barrier (BBB) and diffuse axonal injuries. Secondary injuries, which develop over time, can lead to hypoxia, excessive inflammatory responses and elevated intracranial pressure [7,8]. The diagnosis of TBI is complex and involves comprehensive assessment of medical imaging, clinical examination and laboratory analyses. Clinically, in addition to the Glasgow Coma Scale, the Injury Severity Score (ISS) can be used to assess trauma severity in patients with multiple injuries. The ISS is calculated by assigning scores to the three most severely injured body regions. In detail, the ISS scores range from 0 to 75. ISS scores between 16 and 24 are classified as severe, whereas a score higher than 25 indicates very severe trauma [9].

The protein S100B can be quantified in patient serum as a marker of brain tissue damage, with elevated levels correlating with abnormalities observed on CT imaging [10]. S100B is a member of a Ca^2+^-binding protein family, predominantly expressed by mature astrocytes but present in oligodendrocytes and other cells belonging to the central nervous system [11]. Generally, S100B is involved in calcium homeostasis, cell differentiation and the cell cycle [12]. Upon TBI, extensive injury of neurons causes activation of astrocytes, leading to the secretion of S100B. While there is a disruption of the BBB, S100B can be released systemically into the serum. Therefore, the S100B serum concentration correlates with the extent of brain tissue damage and is considered a useful biomarker for TBI diagnosis and progression [13,14,15]. Moreover, the cascade of primary and secondary injury mechanisms of TBI can give rise to further pathological processes, particularly the development of heterotopic ossification (HO). HO is defined as the abnormal formation of bone tissue outside the skeletal system, typically occurring in soft tissues [16]. HO most frequently manifests in major joints, such as the shoulder, elbow, or hip, leading to pain, swelling and restricted range of motion [16,17]. The occurrence of HO could result from various causes. It may develop as a complication following orthopedic procedures, such as endoprosthetic surgery, or arise in neurogenic form, particularly after severe TBI. In patients with TBI alongside a polytrauma, including bone fractures or substantial muscle injures, 10–20% develop the neurogenic form of HO. Moreover, clinical studies revealed a sexual dimorphism, which showed that male patients are more frequently affected by HO than female patients [18]. The exact pathogenesis of the neurogenic form of HO is complex and not yet fully understood, since it requires a complicated interplay among cells, secreted cytokines, signaling pathways and a microenvironment that promotes osteogenic differentiation [19]. The formation of HO requires stem or progenitor cells with the capacity to differentiate into the osteogenic lineage, which may be altered by dysregulated processes [20]. Moreover, immune cells, particularly macrophages, may play an essential role in the formation of HO. In a study conducted by Zhang and colleagues, it was revealed that M2 macrophages are able to stimulate osteogenic differentiation of adipose tissue MSCs in vitro. Furthermore, these macrophages can secrete osteoinductive factors, such as Oncostatin M, BMP-2, TGF-β1 and Substance P, which contribute to HO formation [21,22,23]. Additionally, different signaling pathways are involved in the formation of HO, including NF-κB-, BMP/TGF-β- and Wnt/β-catenin signaling [24,25,26,27]. TGF-β1 is another cytokine that may contribute to the development of HO. Following TBI, there is an increase in serum concentrations of TGF-β1, particularly in patients with fractures (e.g., polytrauma patients) [28].

In this study, we collected and characterized sera of male and female TBI patients to analyze potential cellular osteogenic effects. We established a novel human stem cell culture model to evaluate osteoinductive effects of TBI sera contributing to HO formation. For this purpose, we chose two different cell types, adipose-derived stem cells (ADSCs) and human skeletal stem cells (SSCs). The osteoinductive capacity of TBI sera on these two cell types was analyzed, with special consideration of sex-specific differences. In addition, the TGF-β1 serum levels were quantified in male and female TBI patients and compared to those of healthy controls. Finally, the important role of TGF-β1 in HO was analyzed by a neutralization assay.

## 2. Materials and Methods

### 2.1. Characterization of Patients

Following the accident that caused the TBI, the patients were taken to the emergency room at the Protestant Hospital of the Bethel Foundation in Bielefeld. Clinical diagnostics and treatment were conducted in accordance with clinical standards and national guidelines. Thus, the initial clinical GCS and ISS were assessed in the emergency room during the body-check procedure. Blood samples were taken for further analysis, including S100B analysis using the Elecsys^®^ S100 assay (Roche, Basel, Switzerland), and analyzed at the Institute of Laboratory Medicine and Microbiology (Protestant Hospital of Bethel Foundation, Bielefeld, Germany). The Department of Diagnostic and Interventional Radiology (Protestant Hospital of Bethel Foundation, Bielefeld, Germany) conducted radiological emergency diagnostics. All medical procedures were performed by medical specialists.

### 2.2. Collection of Serum Samples from TBI Patients

On each sampling day, a total of 40 mL blood (BD Vacutainer, BD, Plymouth, UK) was collected per patient. Glass tubes were chosen to minimize protein binding to the inner wall of the tubes. Blood collection was performed for each patient between 8:00 and 10.00 a.m. The tubes were allowed to clot for 30 min at room temperature before centrifugation at 1500× *g* for 15 min (Centrifuge 5810 R, Eppendorf, Hamburg, Germany). The resulting serum was carefully removed, aliquoted into low protein binding tubes (Eppendorf, Hamburg, Germany) and either used directly for analysis or stored at −80 °C. Hemolytic serum was discarded. On average, 15–20 mL of serum was obtained from each patient per collection. To assess cytotoxic effects of the sera on SSCs, the samples were heat-inactivated in a water bath at 56 °C for 30 min.

### 2.3. Isolation and Characterization of Human Skeletal Stem Cells

Human skeletal stem cells (SSCs) were extracted from femoral heads of patients who received total hip arthroplasty surgery performed by the Department of Trauma Surgery and Orthopedics of the Protestant Hospital of Bethel Foundation. The experimental procedures were ethically approved by the responsible ethics commission (ethical commission Westfalen-Lippe No. 2019-704-f-S). Spongiosa material and trabecular bone marrow were extracted from the femoral head using a Luer instrument. Continuing this process, SSCs were isolated by mechanical disintegration with a scalpel followed by enzymatic digestion using 250 U/mL collagenase type II (Worthington Biochemical, Lakewood, NJ, USA). Finally, the cells were separated from the remaining material by density gradient centrifugation. Isolated SSCs were resuspended in culture medium containing DMEM/F12 (PAN Biotech, Aidenbach, Germany), 2% human platelet lysate (HPL, STEMCELL Technologies, Vancouver, BC, Canada), 1% penicillin/streptomycin (Sigma Aldrich, St. Louis, MO, USA), 2 mM L-glutamine (Sigma Aldrich, St. Louis, MO, USA) and heparin (Sigma Aldrich St. Louis, MO, USA), and seeded in a T25 flask (Sarstedt, Nümbrecht, Germany). After reaching high confluency in passage 0, cells were prepared for flow cytometry and characterized using our previously described method [29].

### 2.4. Cell Culture

The work involving primary cells was conducted within biological safety cabinets, according to safety protocols for sterile procedures. The experiments were conducted utilizing SSCs isolated from a total of four male and three female donors, as indicated in Table 1.

Isolated SSCs were seeded and grown in T25 cell culture flasks, precoated with 0.1% gelatin, in culture medium consisting of DMEM/F12, 1% penicillin/streptomycin, 2 mM L-glutamine, 3 U/mL heparin and 2% HPL. Cells were fed every two to three days by complete replacement of culture medium. The SSCs were cultivated in a humidified incubator (CB150, Binder, Tuttlingen, Germany) set in a controlled atmosphere of 5% CO_2_ and a temperature of 37 °C. Passaging was performed utilizing trypsin/EDTA (Sigma Aldrich, St. Louis, MO, USA) after complete removal of culture medium and washing with PBS (PAN Biotech, Aidenbach, Germany). When the cells were detached, the cell suspension was centrifuged at 300× *g* for 5 min. Subsequently, supernatant was discarded and formed cell pellets were resuspended with culture medium. Finally, cells were seeded in the required amount for further experiments.

Human adipose derived stem cells (ADSCs) were acquired from Lonza (PT-5006, Lonza Group, Basel, Switzerland) from one male and one female donor, as shown in Table 2.

ADSC cultivation medium consisted of DMEMF/12, 10% fetal calf serum (FCS, Sigma Aldrich, St. Louis, MO, USA), 1% penicillin/streptomycin and 2 mM L-Glutamine.

To evaluate cytotoxic effects of TBI serum on SSCs, cells were cultured for seven days and subsequently stained with trypan blue (Sigma Aldrich, St. Louis, MO, USA) to distinguish between viable and non-viable cells.

### 2.5. Osteogenic Differentiation

In order to induce osteogenic differentiation in vitro, the wells used for SSCs were initially coated with liquid collagen and subsequently with collagen type I fibers. The collagen fibers were isolated using a method that was reported previously [30]. SSCs were seeded onto this collagen scaffold at a density of 5 × 10^3^ cells and subsequently incubated in a humidified incubator at 37 °C and 5% CO_2_. ADSCs were seeded at 5 × 10^3^ cells per untreated wells. After reaching high confluency, cultivation medium was discarded and replaced with an osteoinductive medium (OIM) composed of DMEM high glucose (PAN Biotech, Aidenbach, Germany), 10% fetal bovine serum (FBS, Sigma Aldrich, St. Louis, MO, USA), 1% penicillin/streptomycin, 2 mM L-glutamine, 100 nM dexamethasone (Sigma Aldrich, St. Louis, MO, USA), 10 mM sodium β-glycerophosphate (Sigma Aldrich, St. Louis, MO, USA) and 2.5 mM ascorbic acid 2-phosphate (Sigma Aldrich, St. Louis, MO, USA). Depending on the experimental approach, the OIM was supplemented with 10% serum obtained from patients with severe TBI. SSCs were cultured with OIM for a total duration of either 7 or 14 days, while ADSCs were cultivated for 21 days, with the OIM being replaced every 3 days.

Furthermore, a neutralization assay was conducted to assess the impact of TGF-β1 on osteogenic differentiation capacity. For this instance, SSCs were treated according to the previously described differentiation method. The OIM was supplemented with 40 µg/mL, 20 µg/mL or 10 µg/mL of a TGF-β1 neutralizing antibody (chicken, R&D Systems, Minneapolis, MN, USA, AF-101-NA) and 10% TBI serum. The SSCs were differentiated for a total duration of 14 days.

Osteogenic differentiation potential was evaluated using Alizarin Red S (ScienCell, Carlsbad, CA, USA) staining, which allows for the detection of calcium depositions, indicating successful osteogenic differentiation. The staining procedure was carried out according to the manufacturer’s instructions. Subsequently, acetic acid was used to extract Alizarin Red S in order to determine its concentration by photometry (Tristar^2^ S LB 942, Berthold Technologies, Bad Wildbad, Germany), with absorbance measured at 405 nm [31]. Quantification of Alizarin Red S was performed by preparing triplicates for each sample.

### 2.6. Determination of TGF-β1 Serum Levels

Concentration levels of TGF-β1 were measured immediately after blood collection using a sandwich ELISA assay (Human/Mouse TGF beta-1 Uncoated ELISA Kit, Thermo Fisher Scientific, Waltham, MA, USA), in line with the instructions provided by the manufacturer. A 1:25 dilution of serum samples was prepared to reduce potential interference from matrix effects. Additionally, samples were incubated overnight at 4 °C in order to achieve the highest sensitivity. All samples were incubated and measured in duplicates using the microplate reader Tristar^2^ S LB 942 (Berthold Technologies, Bad Wildbad, Germany).

### 2.7. Statistical Analysis

All statistically processed data were tested initially for normal distribution using the Shapiro–Wilk test. The unpaired *t*-test was employed to analyze two normally distributed groups. When examining multiple groups, an ordinary one-way ANOVA was performed when data were normally distributed, or the Kruskal–Wallis test was utilized if data were not normally distributed. Statistical analysis was carried out using GraphPad Prism 10.4.1 (GraphPad Software, Boston, MA, USA).

## 3. Results

### 3.1. Study Design

**Figure 1:**
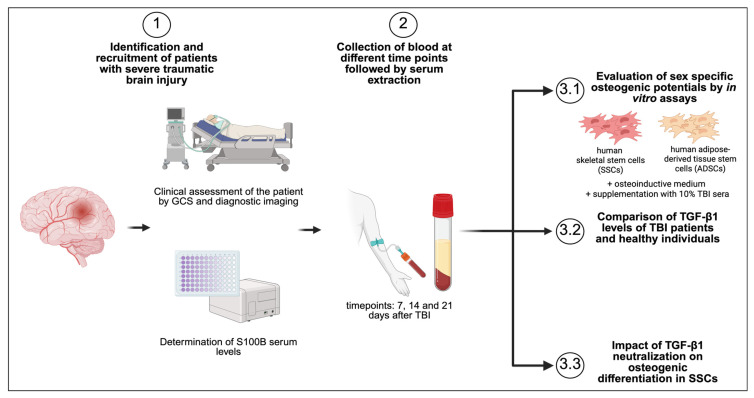
Diagram of the study design. Briefly, patients were recruited based on a combination of clinical assessment and diagnostic imaging, and measurement of initial S100B serum levels. Continuing, blood was collected 7, 14 and 21 days from patients matching the requirements of this study. Initially, sera of the patients were utilized to investigate the impact on the osteogenic differentiation potential in human skeletal stem cells (SSCs) and adipose-derived stem cells (ADSCs). Following this, TGF-β1 levels of TBI patients and healthy individuals were compared. Lastly, the effect of TGF-β1 on the osteogenic differentiation potential was confirmed by neutralization assays in SSCs. Created in BioRender. Kaltschmidt, C. (2025). https://BioRender.com/3prloly.

The general study design is illustrated in Figure 1. Serum samples were obtained from four male and four female patients diagnosed with severe TBI. Samples were collected at 7, 14 and 21 days post-injury during patient treatment. Only patients with GCS scores between 3 and 8 and S100B serum levels greater or equal than 0.1 µg/L were included. Additionally, serum samples from 10 healthy male and 13 healthy female donors were collected and used as controls for TGF-β1 measurements.

### 3.2. Clinical and Laboratory Characterization of TBI Patients

All patients included in this study, with male patients shown in Table 3 and female patients shown in Table 4, primarily suffered a severe traumatic brain injury. Patients were involved in different incidents, including vehicle accidents and falls from a height. Clinical assessment was performed by scoring the patients on the GCS and laboratory analysis of S100B serum concentration levels. Elevated initial S100B concentrations were detected in all sera derived from male TBI patients. More specifically, all male patients exceeded the S100B cut-off value of 0.1 µg/L [32], with a peak concentration of 3.930 µg/L observed in patient TBI M4. Furthermore, all male donors exhibited a clinical GCS score below 8. In addition, ISS was assessed for each patient, indicating all male patients were severely injured by exceeding a score of 25 (Table 3).

The female patients involved in this study also experienced severe traumatic brain injuries caused by falls or traffic accidents. Similar to the male patients, the female patients demonstrated increased S100B serum levels, with a maximum concentration of 7.210 µg/L in patient TBI F3. All female patients were evaluated with a score of 3–7 on the GCS during initial clinical assessment, signifying serious damage to the brain tissue (Table 4). Assessing ISS for the female patients indicates severe injuries, with a score lower than their male counterparts.

In summary, both male and female patients exhibited biochemical and clinical indicators consistent with severe TBI.

**Figure 2:**
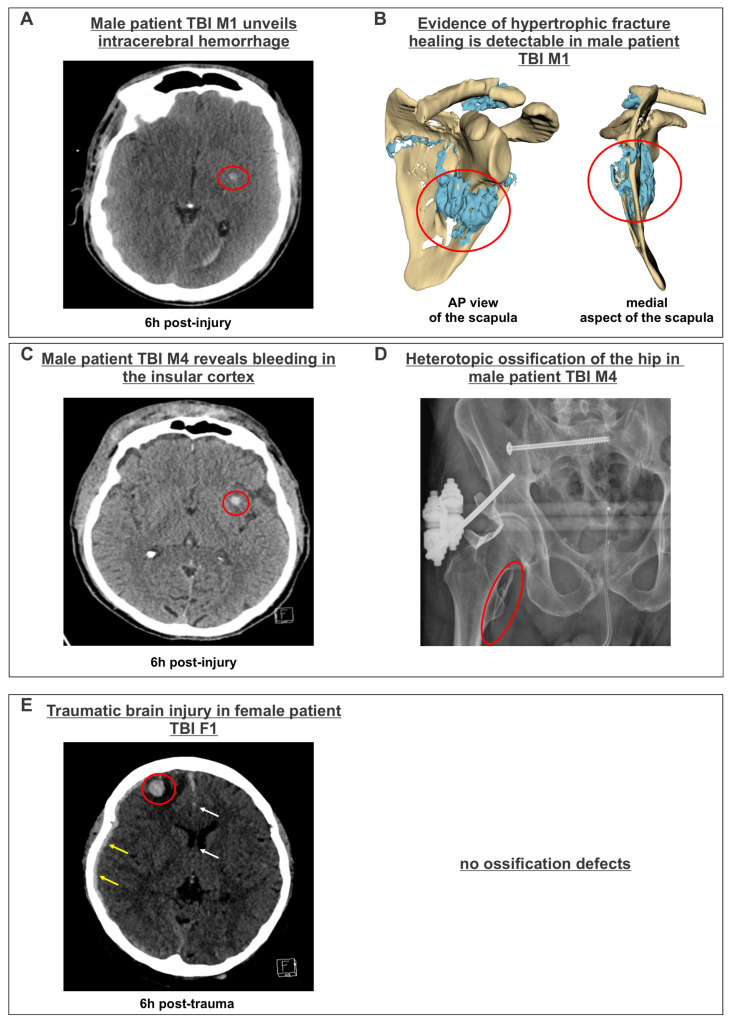
Analysis of radiological imaging in male and female TBI patients. (**A**) CT slide of male patient TBI M1 depicting an intracerebral hemorrhage in the centrum semiovale (red circle) six hours post-trauma. (**B**) The 3D-CT reconstruction of a scapular fracture from patient TBI M1 3 weeks pI, revealing ectopic bone tissue build through hypertrophic fracture healing (blue tissue within red circle) in the anterior–posterior (AP) view and medial plane. (**C**) CT slide of male patient TBI M4 showing a hemorrhage in the insular cortex (red circle) six hours post-trauma. (**D**) X-ray image of the hip of patient TBI M4 revealing a thorn-shaped ectopic bone formation (red circle) resulting from heterotopic ossification. (**E**) CT scan of female patient TBI F1 unveiling frontal contusional hemorrhage (red circle), an acute subdural hematoma (yellow arrows) and a leftward midline shift (white arrows) six hours post-trauma.

The assessment of the patients’ radiological diagnostics identified multiple findings regarding the brain tissue damage that correlated with the clinical diagnostics. Computer tomography (CT) of the head revealed a hemorrhage in the centrum semiovale (red circle, Figure 2A), along with a subarachnoid hemorrhage in the cisterna interpeduncularis in male patient TBI M1. These hemorrhages did not induce a midline shift six hours post-injury. Subsequent imaging diagnostics after 3 weeks indicated an abnormality in the ossification process of a scapular fracture in patient TBI M1. More precisely, the formation of irregular bone mass beneath the fracture, resulting from hypertrophic fracture healing, was identified (red circles, Figure 2B). Furthermore, male patient TBI M4 had also sustained a bleeding in the insular cortex (red circle, Figure 2C). In this patient, the development of ectopic bone resulting from heterotopic ossification was identified using radiological scans after 4 weeks. In detail, a thorn-shaped structure of ectopic bone was observed in the hip of male patient TBI M4 (red circle, Figure 2D; Table 3). While patient TBI M2 had a milder variant of hypertrophic fracture healing in the pelvis, no anomalies in ossification processes were observed in patient TBI M3. The radiological examination of the female TBI patients revealed different forms of hemorrhages in all female patients, with patient TBI F1 being diagnosed with an acute subdural hematoma (yellow arrows), a frontal contusional hemorrhage (red circle) and a consequent midline shift six hours post-trauma (white arrows) (Figure 2E). Contrary to their male counterparts, no defects in ossification processes were observable in female TBI patients after three weeks (Table 4). Since the sera of male and female TBI patients were collected during treatment, the administered medications were also examined. Overall, almost all patients were sedated with various medications (propofol and sufentanil). In addition, nearly all patients, except TBI F2, received metamizole. Furthermore, many patients developed infectious complications during intensive care and were treated with different antibiotics (cefotaxime, cefuroxime, ciprofloxacin and vancomycin). To support circulation, almost all patients, with the exception of TBI F1, received norepinephrine in different doses. All medications administered during the study period are listed in Supplementary Tables S1–S8.

### 3.3. Treatment of Human Skeletal Stem Cells with TBI Serum Revealed No Cytotoxic Effects

**Figure 3:**
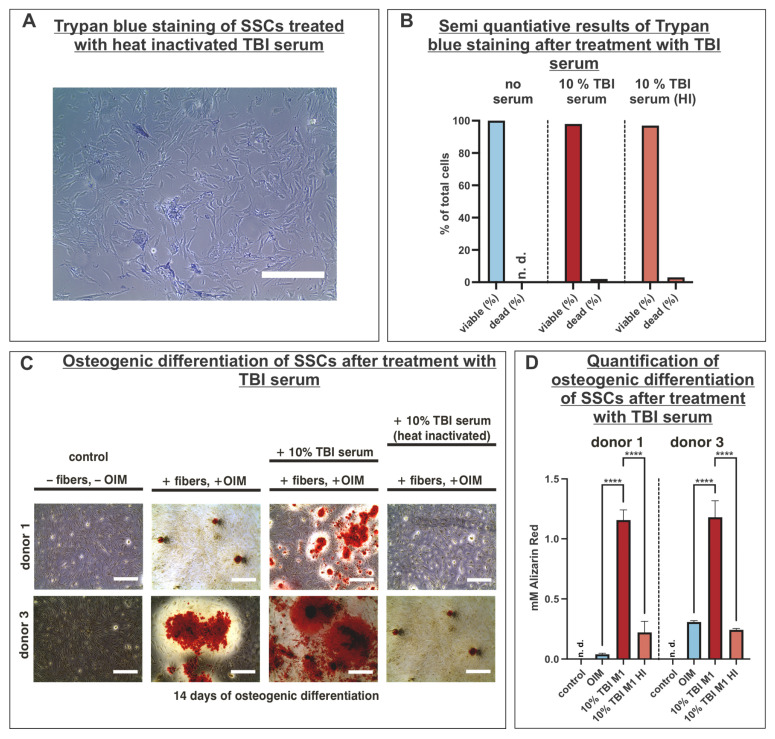
Heat-inactivated (HI) TBI serum did not alter SSC viability but led to a substantial decrease in osteogenic differentiation potential. (**A**) Trypan blue staining of donor 1 cultured in medium supplemented with heat-inactivated TBI serum. (**B**) Semiquantitative analysis of viable and dead cells followed by cultivation in medium supplemented with no serum, heat- and non-heat- inactivated TBI serum. (**C**) Analysis of osteogenic differentiation capacity by Alizarin Red staining with heat- and non-heat-inactivated TBI serum in donors 1 and 3. (**D**) Quantification of Alizarin Red staining revealed significantly decreased osteogenic differentiation capacity when TBI serum was heat-inactivated. Data are shown as mean ± SEM, ordinary one-way ANOVA; **** *p* < 0.0001 was considered significant. Scale bars in images (**A**,**C**) represent 400 µm. n.d.: not detectable.

To determine if the supplementation of 10% TBI serum M1 to the cultivation medium had a cytotoxic effect on SSCs, the cells were cultured with cultivation medium supplemented with either 10% TBI serum M1 or 10% heat-inactivated TBI serum M1 for seven days. The results of trypan blue staining indicated no differences between the control group (cultivation medium alone; 100% viable cells) and the groups treated with either 10% TBI serum (98% viable cells vs. 2% dead cells) or 10% of the heat-inactivated serum (97% viable cells and 3% dead cells) and cultivation medium (Figure 3A,B). Next, it should be examined if the heat inactivation of the TBI serum affected osteogenic differentiation. SSCs derived from donors 1 and 3 were differentiated for 14 days with OIM supplemented with 10% of non- and heat-inactivated TBI serum. In the control condition without the addition of OIM or cultivation on collagen fibers, no osteogenic differentiation potential was detected by Alizarin Red staining (Figure 3C,D). Following this result, it was indicated for both donors that the osteogenic differentiation potential was significantly diminished when the serum was heat-inactivated compared to the approaches with the non-heat inactivated serum, resulting in the denaturation of protein components that may affect osteogenic differentiation capacity (Figure 3C,D). Moreover, it was evident that the addition of TBI serum to OIM resulted in a substantial increase in the Alizarin Red S concentration compared to OIM alone, indicating enhanced osteogenic differentiation capacity (Figure 3C,D). In summary, TBI serum did not cause cytotoxicity in SSCs, but heat inactivation of serum decreases osteogenic differentiation potential significantly. Therefore, non-heat-inactivated sera were applied to the cells for the following experiments.

### 3.4. Supplementation of Osteoinductive Medium with TBI Sera Markedly Enhances Osteogenic Differentitation Potential of Male Adipose-Derived Stem Cells

**Figure 4:**
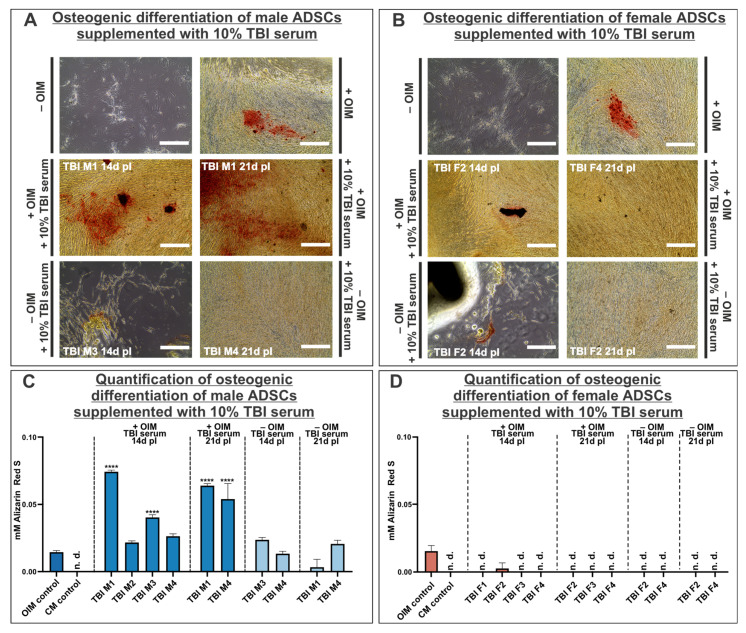
Male ADSCs exhibited enhanced osteogenic differentiation potential under the influence of TBI sera. (**A**) Representative microscopic images of Alizarin Red staining of male ADSCs osteogenically differentiated under the influence of male TBI sera. Male ADSCs (donor 7) underwent osteogenic differentiation in OIM supplemented with TBI sera, leading to calcified areas (depicted in red by Alizarin Red staining). (**B**) Representative microscopic images of Alizarin Red staining of female ADSCs osteogenically differentiated under the influence of female TBI sera. Mineralization in female ADSCs (donor 8) was only detectable when these cells were cultivated in OIM (depicted in red by Alizarin Red staining). (**C**) Quantification of mineralization in male ADSCs demonstrated significantly higher osteogenic potential when OIM was supplemented by sera derived from patients TBI M1, M3 and M4. (**D**) Quantification of mineralization in female ADSCs revealed that supplementation of OIM with TBI sera did not enhance osteogenic differentiation capacity. All approaches involving male and female TBI sera were statistically assessed with an ordinary one-way ANOVA relative to the control group (corresponding OIM control); **** *p* < 0.0001 was considered significant. Scale bars in images (**A**,**B**) represent 400 µm. Data are shown as mean ± SEM. n.d.: not detectable; OIM: osteoinductive medium; CM: cultivation medium; pI: post-injury.

In the initial in vitro cellular assays, we examined the influence of TBI sera on the osteogenic differentiation capacity of adipose-derived stem cells (ADSCs), as this cell type can be mobilized after traumatic injuries and may contribute to the formation of heterotopic ossification. For this purpose, ADSCs underwent osteogenic differentiation for three weeks in osteoinductive medium (OIM), supplemented with 10% TBI sera. The results of the male ADSCs cultivated in OIM supplemented with TBI sera demonstrated an increased osteogenic differentiation capacity compared to those differentiated only in OIM. Interestingly, ADSCs differentiated in OIM supplemented with serum from patient TBI M4 seven days post-injury (pI) exhibited a significantly higher osteogenic differentiation capacity compared to the OIM control, an effect observed only in this condition. Moreover, analysis of the sera obtained 14 days pI revealed that sera from patients TBI M1 and M3 showed a significantly elevated osteogenic differentiation capacity. In the sera obtained from the two patients suffering hypertrophic fracture healing (TBI M1) and heterotopic ossification (TBI M4) 21 days pI, a substantially enhanced differentiation potential was also observed compared to the OIM control. In addition, it should be investigated if the sole application of TBI sera is sufficient to induce osteogenic differentiation in vitro, promoting the differentiation of male ADSCs in culture medium supplemented with TBI sera. Slightly elevated Alizarin Red S concentrations were determined in these approaches compared to the OIM control, but these did not differ significantly from the OIM control (Figure 4A,C). In contrast to the increased osteogenic differentiation potential through supplementation with male TBI sera, it was evident that the addition of female TBI sera had no effect on the osteogenic differentiation ability of the female ADSCs. More precisely, only the serum from patient TBI F2, collected 14 days pI, revealed a minimal quantity of Alizarin Red S, which was much lower than the values of the OIM control (Figure 4B,C).

### 3.5. Supplementation of TBI Sera in Osteoinductive Medium Significantly Promotes Osteogenic Differentiation in Male and Female SSCs

**Figure 5:**
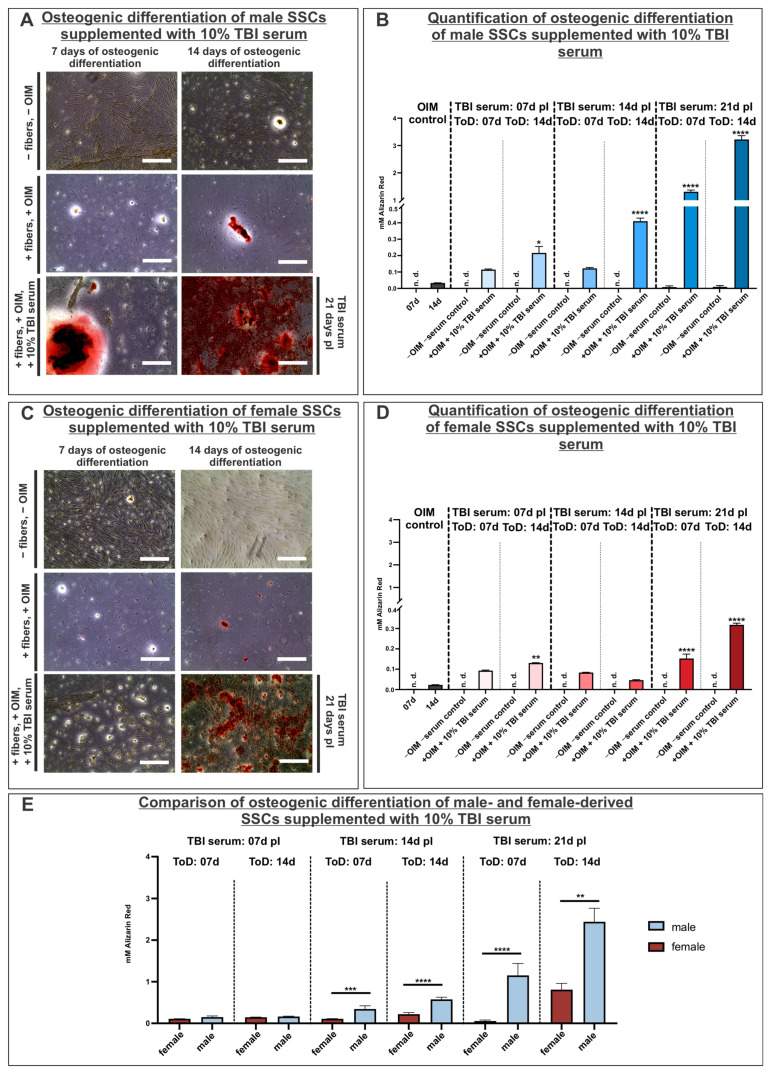
Male and female SSCs revealed enhanced osteogenic differentiation potential in response to TBI sera. (**A**) Osteogenic differentiation of SSCs derived from male donor 2 differentiated for 14 days with the supplementation of TBI serum 21 days pI led to calcified areas, indicating a high osteogenic differentiation potential in comparison to OIM control (depicted in red by Alizarin Red staining). (**B**) Quantification of calcium depositions in male SSCs from donor 5 after 7 and 14 days of differentiation with TBI serum collected 7, 14 and 21 days pI. Data are shown as mean ± SD. All samples supplemented with male TBI serum were statistically assessed with an ordinary one-way ANOVA relative to the control group (14 d OIM control); * *p* < 0.0332, **** *p* < 0.0001 was considered significant. (**C**) SSCs from female donor 5 differentiated for 14 days with the supplementation of TBI serum 21 days pI resulted in calcified areas (depicted in red by Alizarin Red staining). (**D**) Quantification of calcium depositions in female SSCs from donor 5 after 7 and 14 days of differentiation with TBI serum collected 7, 14 and 21 days pI. Data are shown as mean ± SD. All samples supplemented with female TBI serum were statistically assessed with an ordinary one-way ANOVA relative to the control group (14 d OIM control); ** *p* < 0.0021, **** *p* < 0.0001 was considered significant. (**E**) Comparative analysis of osteogenic potential between male (*n* = 3) and female (*n* = 3) SSCs in response to TBI serum. Male SSCs showed significantly higher calcium depositions at 14 and 21 days pI, particularly after 14 days of differentiation. Data are shown as mean ± SEM. Statistical comparisons between male and female groups were performed using the Kruskal–Wallis test; ** *p* < 0.0021, *** *p* < 0.0002, **** *p* < 0.0001 was considered significant. Scale bars in (**A**,**B**) represent 400 µm. ToD: time of differentiation; n.d.: not detectable.

To examine a more specialized stem cell type that may affect the development of heterotopic ossification, human skeletal stem cells (SSCs) from male and female donors were osteogenically differentiated in the presence of TBI serum collected at 7, 14 and 21 days pI. SSCs derived from male donor 2 exhibited slightly enhanced mineralization after 7 days of differentiation with serum collected 7 and 14 days pI, although these effects were not statistically significant compared to the OIM control. In contrast, serum collected at 21 days pI when used for 7-day differentiation had a significantly promoted effect on the osteogenic differentiation capability of the male SSCs in comparison to the OIM control. Throughout the 14-day differentiation of male SSCs derived from donor 2, the Alizarin Red S concentration was significantly elevated at each serum time point (7, 14 and 21 days pI) compared to the OIM control, indicating high osteogenic differentiation potential. Generally, the trend indicated a successive rise, peaking when the SSCs were incubated with the TBI serum collected 21 days pI. Negative control cultures, in which SSCs were maintained without OIM or serum, exhibited no or minimal calcium deposition after both 7 and 14 days of incubation (Figure 5A,B). The osteogenic differentiation capacity of SSCs derived from male donors 3 and 4 also showed a significant increase, particularly in SSCs treated with TBI serum obtained after 21 days (Figures S1 and S2). Comparable results in enhancing the osteogenic differentiation potential have been observed in female SSCs from donor 5. In the female SSCs differentiated for 7 days in response to TBI serum, a notable rise in calcification could only be detected when using the serum collected 21 days pI. After 14 days of directed osteogenic differentiation, a significantly enhanced osteogenic differentiation capacity was identified when SSCs were cultivated in the presence of sera collected 7 and 21 days pI. More specifically, the highest osteogenic differentiation potential was determined when utilizing serum collected 21 days pI and differentiated for 14 days (Figure 5C,D), as already identified in male SSCs. Moreover, increased osteogenic differentiation potential was observed in female SSCs derived from donor 6 when SSCs were incubated with TBI sera obtained after 14 and 21 days for the 14 days of the differentiation period (Figure S3). The same trend was observable in SSCs from female donor 7; in this case, the osteogenic differentiation capacity was significantly enhanced when cells were incubated with TBI serum obtained after 21 days (Figure S4). Overall, it was determined that the osteogenic differentiation capacity was higher in male SSCs. To verify this, data of all male and female cell donors were analyzed cumulatively. Combined analysis of all male (*n* = 3, donors 2, 3 and 4) and female cell donors (*n* = 3, donors 5, 6 and 7) revealed that male TBI serum induced significantly higher osteogenic differentiation capacity compared to female TBI serum, particularly at the 14- and 21-day pI time points. Notably, male TBI serum induced detectable calcium deposition already after 7 days of differentiation, indicating a more rapid and potent osteoinductive effect (Figure 5E).

### 3.6. Investigation of TGF-β1 as a Driver of Heterotopic Ossification: Increased TGF-β1 Serum Concentration Levels in Male TBI Patients

**Figure 6:**
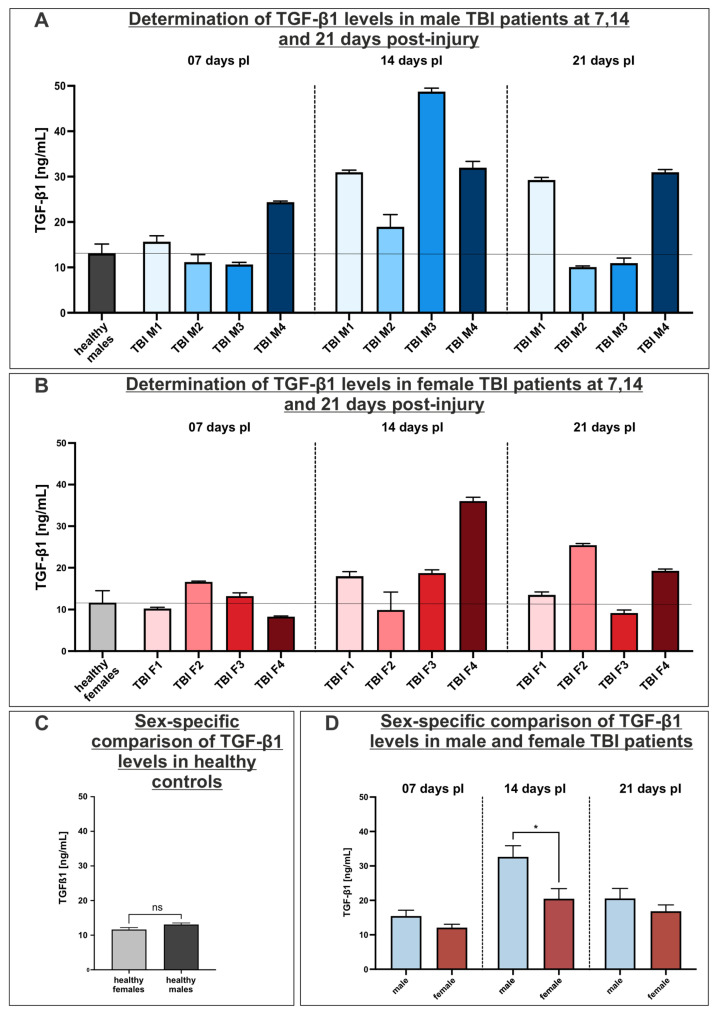
Assessment of TGF-β1 concentration levels in sera from male (**A**) and female (**B**) TBI patients and healthy controls. (**A**) Male TBI patients showed increased TGF-β1 concentrations in comparison to healthy controls, with peaking concentrations 14 days pI. (**B**) Female TBI patients showed increased TGF-β1 concentrations in comparison to healthy controls, with peaking concentrations 14 days pI. (**C**) Healthy controls showed no sex-specific differences in TGF-β1 serum concentrations. Unpaired *t*-test. *p* > 0.05 was considered as not significant (ns). (**D**) Comparison of TGF-β1 serum levels in male and female TBI patients 7, 14 and 21 days pI revealed higher concentration levels of TGF-β1 14 days pI in male TBI patients. Unpaired *t*-test. * *p* < 0.0332 was considered significant. All data shown as mean ± SEM.

To determine the effect of the cytokine TGF-β1, which may be increased in serum following TBI, favoring the development of heterotopic ossification, a sandwich ELISA was performed with the sera derived from male and female TBI patients at 7, 14 and 21 days post-injury (pI). Initially, the TGF-β1 serum concentrations of 13 healthy female and 10 healthy male donors were assessed. The average concentration was determined to be 11.65 ng/mL for female donors and 13.09 ng/mL for male donors (indicated by the horizontal line in Figure 6A–C).

The concentration of TGF-β1 in male TBI-patients, measured 7 days pI, revealed an increased level in donor TBI M4 when compared to the control group (healthy males). Subsequent samples obtained 14 days post-trauma demonstrated a substantial elevation in the TGF-β1 concentration among all male TBI patients. Moreover, the peak concentration of TGF-β1, measured at 48.75 ng/mL, was detected in the male patient TBI M3. Particularly, patient TBI M1 exhibited a significant increase, with TGF-β1 levels doubling within one week. In the TGF-β1 concentration analysis conducted 21 days pI, only male TBI patients M1 and M4 had significantly elevated values compared with the control group. In addition, TGF-β1 concentrations of male TBI patients M2 and M3 neared the concentration levels of TGF-β1 in the control group again at this time (Figure 6A).

A similar trend in the concentration levels of TGF-β1 was also observed in the analysis of the female TBI patients. Seven days post-injury, a slight increase in TGF-β1 was observed just in patient TBI F1. The peak concentrations of TGF-β1 were determined in the female TBI patients 14 days post-trauma. Female patients TBI F1, F3 and F4 exhibited substantially higher TGF-β1 levels in the 14 days post-trauma. At this timepoint, the maximum concentration of 36.03 ng/mL was measured in patient TBI F4. After 21 days of the TBI, TGF-β1 concentrations in patients TBI F1 and F3 returned to the levels of healthy female donors. However, elevated concentration values were detected in patients TBI F2 and F4 (Figure 6B).

A comparison of the results of the healthy controls and patients by sex indicates that no statistically significant differences between the healthy males and females were detectable, while male patients exhibited a higher average TGF-β1 concentration compared to their female counterparts (Figure 6C). Particularly, at the timepoint 14 days post-injury, male serum concentrations of TGF-β1 were significantly elevated in comparison to their female patients (Figure 6D).

### 3.7. Neutralization of TGF-β1 Strongly Reduces Osteogenic Differentiation Capacity in Male SSCs

**Figure 7:**
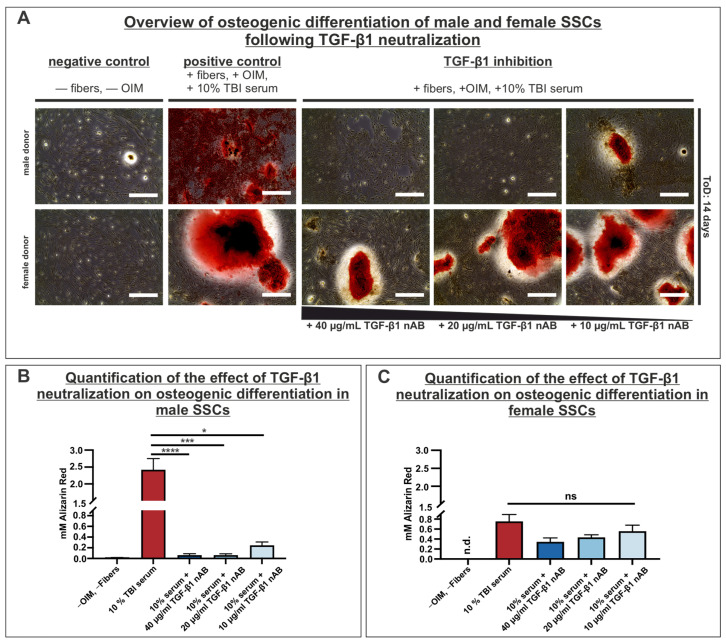
Inhibition of TGF-β1 led to highly decreased osteogenic differentiation capacity in male SSCs, whereas female SSCs revealed no significant reduction. (**A**) Representative microscopic overview of Alizarin Red staining in the negative control, positive control and TGF-β1 inhibition tests utilizing a TGF-β1 neutralizing antibody (nAB). The scale in the microscope images equals 400 µm. (**B**) Utilization of a TGF-β1 nAB highly decreased osteogenic differentiation capacity in male SSCs (*n* = 3) in all conditions. (**C**) Osteogenic differentiation capacity was not statistical significantly affected by applying a TGF-β1 nAB in female SSCs (*n* = 3) in all neutralization conditions. Data shown as mean ± SEM. Kruskal–Wallis test; * *p* < 0.0332, *** *p* < 0.0002, **** *p* < 0.0001 was considered significant, *p* > 0.05 was considered as not significant (ns). ToD: time of differentiation; nAB: neutralizing antibody.

In order to assess the effect of TGF-β1, which was found to be elevated in the sera of male and female TBI patients, on the osteogenic differentiation capacity, a neutralization assay of TGF-β1 was conducted. SSCs derived from three male and three female donors were incubated on collagen fibers, and were supplemented either with OIM and TBI serum or, in another approach, with OIM, TBI serum and a TGF-β1 neutralizing antibody. Cells were differentiated for a total duration of 14 days. The osteogenic potential was evaluated with Alizarin Red staining and subsequent quantification. Upon assessing the Alizarin Red staining, it was evident that the positive control of both male and female SSCs exhibited strongly red-colored areas, indicating the presence of calcium inclusions within the cells, which suggested a high osteogenic differentiation potential, which was also corroborated by the quantification of the Alizarin Red staining for male and female positive controls (Figure 7A–C).

A diminished osteogenic differentiation potential was observed when the neutralizing antibody (nAB) was applied. A significant decrease in Alizarin Red was particularly noticeable in the results for the male SSCs. The addition of 40 µg/mL and 20 µg/mL of nAB resulted in a highly significant decrease in osteogenic differentiation potential in SSCs derived from male donors compared to the positive control. Furthermore, a notable decrease in osteogenic differentiation potential was indicated when only 10 µg/mL of the nAB was added (Figure 7B).

The Alizarin Red staining of the preparations including female SSCs revealed that even the addition of 40 µg/mL of the nAB led to no statistically significant decrease in the osteogenic differentiation capacity. This outcome was also present in the approaches containing 20 µg/mL and 10 µg/mL of the nAB (Figure 7C).

In conclusion, these data demonstrated that the supplementation of OIM with TBI serum showed highly induced osteogenic differentiation capacity. Moreover, in neutralization approaches including male SSCs, the osteogenic differentiation potential was significantly diminished by inhibiting the TGF-β1 signaling pathway across all inhibition approaches. In contrast, neutralization of TGF-β1 in female SSCs demonstrated no statistically significant reducing effect on the osteogenic differentiation capacity, indicating a sex-specific role of TGF-β1 in the differentiation process.

## 4. Discussion

This study investigated sex-specific differences in the osteogenic differentiation of ADSCs and SSCs, induced by sera from male and female TBI patients, with a particular focus on TGF-β1 as an osteoinductive factor contributing to the formation of HO following severe TBI in males. Initially, the Glasgow Coma Scale (GCS) was evaluated for all patients upon arrival at the emergency department. The GCS is a common parameter for testing consciousness and brain function after TBI [3]. All patients, both male and female, exhibited a GCS score equal to or below 7, signifying a severe TBI. These findings were validated through measurement of the serum levels of the astrocyte-derived protein S100B. S100B can serve as an indicator of mortality after TBI [13,33]. Especially, very high initial S100B concentrations up to 48 h post-injury may predict a poor TBI outcome [34]. Furthermore, a correlation was found between the release of the S100B protein and the volume of brain contusions [35,36]. The possible disruption of the BBB may lead to the release of the S100B protein following TBI, with a peak concentration observed approximately six hours post-injury [13]. S100B may also be utilized in the diagnostic algorithm of TBI. When serum concentrations remain below 0.1 µg/L, a CT scan may be omitted, as the probability of clinically relevant TBI is minimal [37,38]. In this study, all patients exceeded the S100B cut-off value significantly, with the highest concentrations measured in male donor TBI M4 (3.390 µg/L) and female donor TBI F3 (7.210 µg/L). Notably, female patients exhibited higher S100B concentrations than their male counterparts, suggesting a greater severity of TBI in women, which was further examined using CT scans. Recent studies report higher S100B serum concentrations in polytraumatized patients than in individuals with isolated brain injuries [39]. These findings may be explained by the extracerebral release of S100B from soft tissue injuries in polytraumatized patients [40]. Interestingly, this pattern was not evident in our cohort: female TBI patients with isolated skull injuries more frequently displayed higher S100B levels. In the larger context of sex-specific differences in TBI, epidemiological data showed that males experience TBI more frequently than females [5,41]. The sex difference likely results from multiple risk factors. Males typically demonstrate higher risk-taking behavior, which elevates the possibility of accidents [42]. Furthermore, males are more likely to work in professions associated with higher risk of head injuries, such as military and construction jobs [43,44]. In addition, males consume more alcohol on average, which may enhance the probability of accidents resulting in TBI [45]. TBI etiology differs by sex, with traffic accidents being more common in males and falls more frequent in females [46]. Additionally, sex significantly affects the outcomes of TBI. A study by Herrera-Melero and coworkers revealed that the mortality rate for intensive care unit admission was elevated for female patients. Furthermore, the mortality rate 6 months post-injury was also increased in female patients compared to their male counterparts [47]. An investigation of Ottochian and colleagues indicated that postmenopausal women had an increased mortality rate after TBI [48]. In conclusion, males are more frequently affected by severe TBI, while females tend to experience a more severe outcome of the TBI [49,50,51,52]. Age is also considered as an important factor in the incidence of TBI. In cases of moderate and severe TBI, the occurrence increases steadily and reaches a peak in individuals older than 90 years. Across all age groups, incidence values are higher for male patients [5]. Within the patient cohort of this study, male patients were, on average, younger than female patients. Another limitation, based on individual diagnoses and the Injury Severity Score, is that male patients generally sustained more severe injuries and were more frequently polytraumatized, whereas female patients more often presented with isolated skull injuries. An ISS score of 16 or higher can be considered a risk factor for the development of HO, and was present in all patients except TBI F1 [53]. In this context, it could be hypothesized that more severe injury patterns indicated by higher ISS scores result in a higher growth factor release, which may promote the development of HO. Since the availability of trauma patients matching our criteria was limited, we could only collect material from four male and four female TBI patients. To further enhance the validity of the findings, it should be considered to have a larger and more balanced patient population. The association between TBI and HO is well established. Epidemiological data of these two diseases are also connected to each other, indicating that the incidence of TBI is also impacting the incidence of HO. While men are more often affected by HO, specific age groups in which HO predominantly occurs has not yet been clearly defined [54]. Moreover, younger patients tend to have a larger pool of stem cells and more active bone remodeling [55,56]. In summary, male sex and younger age can be considered risk factors that may promote the development of HO [19].

Interestingly, this study identified defects in ossification processes that occurred predominantly in male patients. While hypertrophic fracture healing (patients TBI M1 and M2) and heterotopic ossification (TBI M4) after TBI were observed in male patients, none of these abnormalities were identified in female patients. Given that the precise pathophysiology of neurogenic HO remains insufficiently explained, we established a stem cell culture model in this study to examine a potential osteoinductive effect of sera derived from male and female TBI patients. The specific cell and stem cell types directly contributing to HO formation remain unclear, although it was hypothesized that these cells originate from mesodermal lineages [57]. In this human stem cell model, two distinct cell lines were analyzed: human adipose-derived stem cells (ADSCs) and human skeletal stem cells (SSCs). ADSCs originate from the mesoderm and exhibit nearly similar characteristics to bone marrow mesenchymal stem cells (bmMSCs) regarding expression patterns and differentiation potential [58,59]. In addition, the isolation process of ADSCs does not require invasive extraction procedures, making it technically less complex and ensuring a higher yield [60]. Furthermore, ADSCs have a higher proliferation capacity compared to bmMSCs [61]. In contrast, SSCs were selected due to their multipotency, rapid differentiation capability to the osteogenic fate and presence in a specialized stem cell niche within the bone [29,62]. Moreover, SSCs are frequently found in fracture calluses responsible for fracture repair [63]. The first step to establish this cell culture model was to investigate the potential cytotoxic effects of the sera on cells. It should be evaluated whether the heat inactivation of the sera is essential to denature proteins, especially complement proteins, which could cause allogenic damage to the utilized cells [64]. The addition of heat-inactivated serum to the culture medium did not affect the growth of the SSCs. Moreover, the effect of heat-inactivated serum supplementation in OIM on the osteogenic differentiation capacity was also examined. A substantial decrease in the differentiation potential of the SSCs was identified. Heat inactivation appears to induce denaturation of serum proteins, thereby significantly affecting the osteogenic differentiation capacity of SSCs. This indicates that serum proteins play a central role in mediating the osteogenic induction.

The examination of TBI sera in a cellular model for HO revealed the sex-specific enhancement of the osteogenic differentiation capacity, with a markedly stronger effect observed when OIM was supplemented with male TBI sera. ADSCs exhibited a strong osteogenic differentiation in response to male serum, while no differentiation was observed in ADSCs treated with female serum. Contrary, the differentiation results of SSCs supplemented by TBI serum indicated a significant increase in calcification in both male and female SSCs. It is important to note that these values were substantially higher in male SSCs. Comparing these two cell types revealed that the osteogenic differentiation capacity was significantly higher in SSCs. SSCs are highly specialized stem cells capable of osteogenic differentiation within 7 days, whereas ADSCs and bmMSCs require a minimum of 21 days [29,65]. Moreover, osteoinductive substances and growth factors in the sera of both sexes can substantially affect the osteogenic differentiation potential. In this context, the age of the patients from whom serum was collected may be a relevant factor, as concentrations of various growth factors and cytokines are generally higher in younger individuals [66]. When analyzing the effects of the medications administered to patients, it was generally noted that none of these active substances exhibited a positive inducing effect to the osteogenic differentiation, as described in the literature. For propofol and sufentanil, which were administered to almost all patients, no evidence of an influence on osteogenic differentiation has been reported. Likewise, the use of non-steroidal anti-inflammatory drugs, even in combination with pantoprazole or metamizole, did not affect the osteogenic differentiation of MSCs in vitro [67]. In contrast, several active substances were identified that may exert inhibitory effects on stem cells and osteogenic differentiation. Notably, the antibiotics (cefotaxime, cefuroxime, ciprofloxacin and vancomycin) have been reported to impair osteogenic differentiation in a dose-dependent manner, as indicated by the cell number and reduced alkaline phosphatase activity in human osteoblasts in vitro [68,69,70]. The exact antibiotic doses present in the cellular experiments remain uncertain, and since some of the medications have short half-lives (cefuroxime: about 2.1 h; cefuroxime: about 2.5 h; vancomycin: 3 to 9 h), a biological effect on stem cells and osteogenic differentiation can be considered minimal [71,72,73]. The cytokine TGF-β1 may be involved in the formation of HO. Generally, TGF-β1 impacts various biological processes, including cell proliferation, cell survival, cell differentiation, cell migration and the production of the extracellular matrix [74,75]. TGF-β1 signaling acts primarily through SMADs; however, it can also activate mitogen-activated protein kinases (MAPKs) independently of SMADs [76]. In particular, as shown in a murine study, TGF-β1 is able to activate TGF-β–activated kinase 1 (TAK1; also known as MAP3K7), leading to the expression and phosphorylation of Runx2 in downstream signaling [77]. Runx2 is regarded as the master transcription factor essential for osteogenic differentiation in vitro [78]. In a study by Li and colleagues, it was shown that the overexpression of TGF-β1 in bmMSCs led to increased mRNA expression of Osteocalcin and Runx2 [79]. Moreover, TGF-β1 had been identified for its involvement in physiological fracture healing, and reduced TGF-β1 levels during this process are associated with extended and non-union fracture healing [80,81]. In addition, several studies had demonstrated a neuroprotective effect of TGF-β1 following trauma, indicated by elevated TGF-β1 concentrations that enhanced neuronal survival and decreased the apoptosis of affected cells [82,83]. Consequently, TGF-β1 may have a role in the formation of HO. Reinforcing this, a murine study by Wang and colleagues showed that increased levels of active TGF-β1 can recruit mesenchymal progenitor cells and that attenuation of TGF-β1 inhibits the progression of HO formation in male mice [84]. We therefore considered TGF-β1 as an important factor contributing to the development of HO. We demonstrated that TGF-β1 concentrations were significantly elevated in both male and female TBI patients 14 days post-injury. A direct comparison of these cohorts at that timepoint revealed that the concentrations among male patients were significantly higher compared to their female counterparts. Notably, patient TBI M4, who developed heterotopic ossification of the hip, demonstrated markedly elevated TGF-β1 serum levels at all measured time points. Furthermore, these concentrations exceeded those observed in patient TBI M1 (at one time point) and in patient TBI M2 (at all time points), who exhibited hypertrophic fracture healing. An even greater sex-specific difference was identified in the neutralization of TGF-β1 during differentiation experiments supplemented with male TBI serum. The usage of a TGF-β1 neutralizing antibody significantly diminished the osteogenic differentiation capacity of male SSCs. This effect was not detected in female SSCs. A limiting factor of this neutralization assay might be the absence of a protein control, which prevents unspecific interactions from being fully ruled out. Nevertheless, the antibody was used in several studies demonstrating high specificity for TGF-β1. High specificity was indicated by the reduced expression of phosphorylated SMAD3 and decreased levels of TGF-β1, as measured with ELISA, after antibody treatment [85,86]. Additionally, the antibody did not exhibit cross-reactivity with other TGF-ß isoforms [87]. We may have identified a sexual dimorphism that may alter the development of HO in vivo. The formation of ectopic bone in males may be driven by TGF-β1, whereas, in females, an alternative signaling pathway may affect the development of HO. The Wnt/β-catenin signaling is a possible signaling pathway enhancing the formation of HO, particularly in female patients. It contributes to HO development by promoting angiogenesis and enhancing the osteogenic differentiation of MSCs [88]. Two studies have demonstrated that estrogen promotes osteogenic differentiation through the activation of Wnt/β-catenin signaling, as shown in mouse and human periodontal ligament stem cells [89,90]. These findings suggest that Wnt/β-catenin signaling may act as an alternative mechanism for osteoinduction in female patients, leading to the development of HO. A recently published study by de Vasconcellos and colleagues supports our view that TGF-β1 is important for the development of HO, especially in males. These researchers employed different TGF-β1 inhibitors as a novel therapeutic approach treating post-traumatic HO. Investigations of primary mesenchymal progenitor cells isolated from debrided traumatized human muscle tissue treated with ALK5 inhibitors (SB431542 and Galunisertib) and the SMAD3 inhibitor Halufuginone effectively downregulated Runx2 expression. In an additional HO model with male Sprague–Dawley rats, the dose-dependent treatment with SB431542, Galunisertib and Halufuginone resulted in the downregulation of alkaline phosphatase. Subsequent analysis revealed the inhibited expression of Runx2 in rats after the application of Halufuginone and SIS3 (SMAD3 inhibitors) in a dose-dependent manner [91]. Further studies should aim to identify molecular mediators, such as factors that enhance Wnt/β-catenin signaling, which may contribute to the formation of HO in female patients. Consequently, the analysis of serum proteome of TBI patients, particularly in female patients, may reveal sex-specific pathways and molecular targets which enable the development of sex-specific therapy options.

## 5. Conclusions

This study investigated whether sera from severe TBI patients have osteoinductive capabilities that may induce heterotopic ossification. We developed a stem cell model with SSCs and ADSCs, and found that TBI sera did not show any cytotoxic effects on these stem cell types. Supplementing osteoinductive media with TBI sera increased the osteogenic differentiation capacity, especially in male ADSCs. Incubation of TBI sera with SSCs indicated a strong osteogenic differentiation capacity in both sexes, with higher capacity again in male SSCs. Additionally, we identified TGF-β1 as the driving factor contributing to these observed osteogenic effects in males. In addition, we determined that male TBI patients had higher TGF-β1 serum levels 14 days post-injury. Furthermore, TGF-β1 neutralization significantly decreased osteogenic differentiation in male SSCs only, while no effect was determined in female SSCs. In conclusion, TGF-β1 is crucial for the development and progression of HO in male patients, while different signaling mechanisms may be involved in female patients. Analysis of the serum proteome from female TBI patients may reveal novel factors for developing sex-specific therapy against HO.

## Figures and Tables

**Table 1:** Sample ID, sex and age of selected skeletal stem cell (SSC) donors used in this study.

Sample ID	Sex	Age [Years]
Donor 1	Male	62
Donor 2	Male	59
Donor 3	Male	40
Donor 4	Male	27
Donor 5	Female	80
Donor 6	Female	63
Donor 7	Female	57

**Table 2:** Sample ID, sex, age and batch number of selected adipose-derived stem cell (ADSC) donors used in this study.

Sample ID	Sex	Age [Years]	Batch Number
Donor 8	Male	62	22TL2213997
Donor 9	Female	57	23TL198527

**Table 3:** Clinical and biochemical characterization of male TBI patients with indication of sample ID, sex, age, diagnosis, injury severity score (ISS), S100B levels, clinical GCS scores and ossification processes (−: no ossification; ++: moderate ossification; +++: strong ossification).

Sample ID	Sex	Age [Years]	Diagnosis	Injury Severity Score	S100B Levels [µg/L]	Clinical GCS Score	Ossification
TBI M1	Male	34	TBI, cerebral hemorrhagic contusion, chest trauma, extremity trauma	29	1.470	3	+++ (hypertrophic fracture healing)
TBI M2	Male	30	TBI, epidural/subdural and subarachnoid hematoma, intracerebral hemorrhage, chest trauma, pelvic trauma	34	1.550	3	++ (hypertrophic fracture healing)
TBI M3	Male	18	TBI with diffuse axonal injury, scalping injury, chest trauma, spinal trauma	29	1.110	3	−
TBI M4	Male	77	TBI, cerebral hemorrhagic contusion, chest trauma, extremity trauma, spinal trauma	34	3.930	7	+++ (heterotopic ossification)

**Table 4:** Clinical and biochemical characterization of female TBI patients with indication of sample ID, sex, age, diagnosis, injury severity score (ISS), S100B levels, clinical GCS scores and ossification processes (−: no ossification).

Sample ID	Sex	Age [Years]	Diagnosis	Injury Severity Score	S100B Levels [µg/L]	Clinical GCS Score	Ossification
TBI F1	Female	53	TBI, basilar skull fracture (occipital condyle fracture), subdural hematoma, cerebral hemorrhagic contusion	9	5.060	3	−
TBI F2	Female	50	TBI, subdural and epidural hematoma, basilar skull fracture, cerebral hemorrhagic contusion	26	2.750	3	−
TBI F3	Female	63	TBI, cerebral swelling, subdural hematoma	20	7.210	3	−
TBI F4	Female	45	Penetrating TBI with subdural hematoma, basilar skull fracture (os occipitale)	22	1.350	7	−

## Data Availability

The data presented in this study are available upon request from the corresponding author. The data are not publicly available due to ethical and privacy reasons.

## Supplementary Materials

The following supporting information can be downloaded at: https://www.mdpi.com/article/10.3390/cells14191491/s1**.** Figure S1. SSCs derived from male donor 3 revealed enhanced osteogenic differentiation potential in response to TBI sera. Figure S2. SSCs derived from male donor 4 revealed enhanced osteogenic differentiation potential in response to TBI sera. Figure S3. SSCs derived from female donor 6 revealed enhanced osteogenic differentiation potential in response to TBI sera. Figure S4. SSCs derived from female donor 7 revealed enhanced osteogenic differentiation potential in response to TBI sera. Table S1: Overview of medications administered to patient TBI M1 at 7, 14 and 21 days post-injury. Table S2: Overview of medications administered to patient TBI M2 at 7, 14 and 21 days post-injury. Table S3: Overview of medications administered to patient TBI M3 at 7, 14 and 21 days post-injury. Table S4: Overview of medications administered to patient TBI M4 at 7, 14 and 21 days post-injury. Table S5: Overview of medications administered to patient TBI F1 at 7, 14 and 21 days post-injury. Table S6: Overview of medications administered to patient TBI F2 at 7, 14 and 21 days post-injury. Table S7: Overview of medications administered to patient TBI F3 at 7, 14 and 21 days post-injury. Table S8: Overview of medications administered to patient TBI F4 at 7, 14 and 21 days post-injury.

## Abbreviations

The following abbreviations are used in this manuscript:

TBI	traumatic brain injury
GCS	Glasgow Coma Scale
HO	heterotopic ossification
BBB	blood–brain barrier
MSCs	mesenchymal stem cells
ADSCs	adipose-derived stem cells
SSCs	skeletal stem cells
OIM	osteoinductive medium
CT	computer tomography
HI	heat-inactivated
n.d.	not detectable
pI	post-injury
ToD	time of differentiation
nAB	neutralizing antibody
