# Supplementary material for: A Novel Human Stem Cell Culture Model for Severe Traumatic Brain Injury Reflecting Sexual Dimorphism in Heterotopic Ossification
Source: Cells. 2025 Sep 24;14(19):1491. doi: 10.3390/cells14191491 (PMC12524128; doi:10.3390/cells14191491)
Supplement: Supplementary file 1 [file cells-14-01491-s001.zip › cells-3821605-supplementary.pdf]

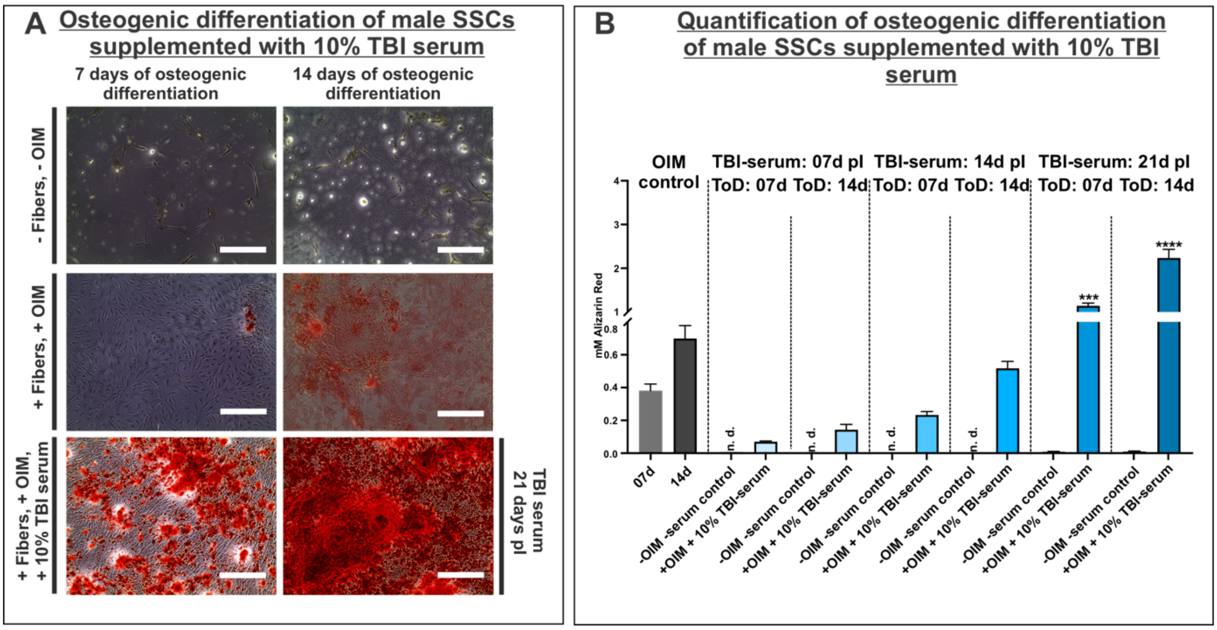

Figure S 1 **SSCs derived from male donor 3 revealed enhanced osteogenic differentiation potential in response to TBI sera.** (A,B) Osteogenic differentiation of SSCs derived from male donor 3 differentiated for 7 and 14 days with the supplementation of TBI serum 21 days pI led to calcified areas indicating a high osteogenic differentiation potential in comparison to OIM control (depicted in red by Alizarin Red staining). Data are shown as mean ± SD. All samples were statistically assessed with an ordinary one-way ANOVA relative to the OIM control group, *** $p < 0.0002$, **** $p < 0.0001$ was considered significant. Scale bars in image A represent 400 µm.

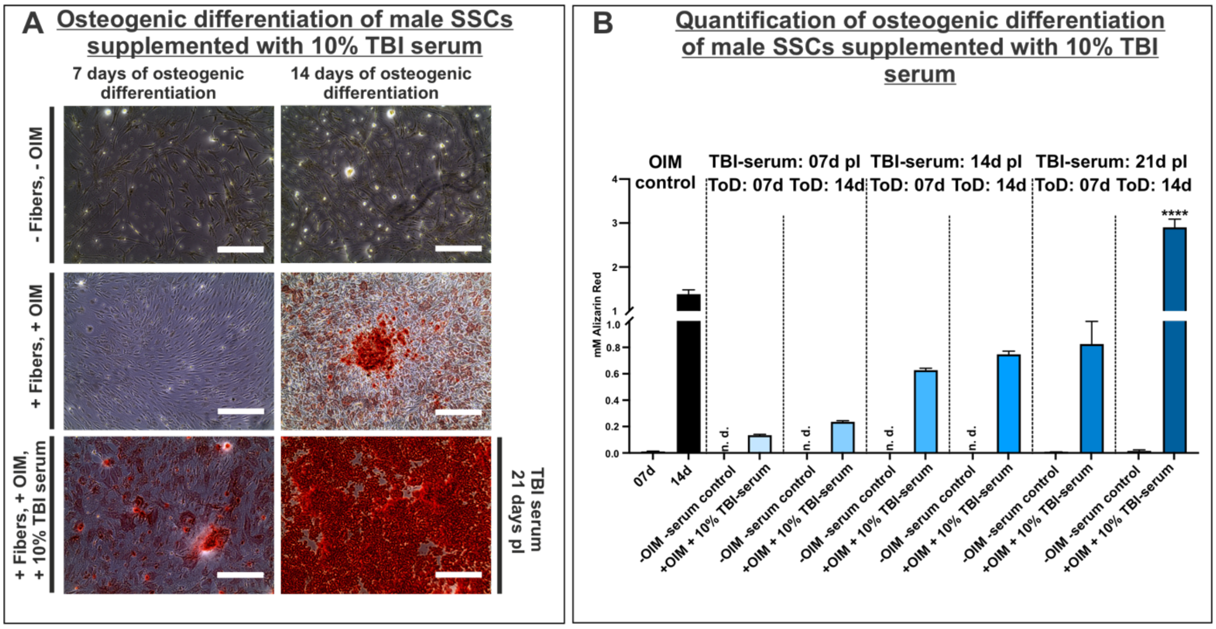

Figure S 2 **SSCs derived from male donor 4 revealed enhanced osteogenic differentiation potential in response to TBI sera.** (A,B) Osteogenic differentiation of SSCs derived from male donor 4 differentiated for 14 days with the supplementation of TBI serum 21 days pI led to calcified areas indicating a high osteogenic differentiation potential in comparison to OIM control (depicted in red by Alizarin Red staining). Data are shown as mean ± SD. All samples were statistically assessed with an ordinary one-way ANOVA relative to the OIM control group, **** $p < 0.0001$ was considered significant. Scale bars in image A represent 400 µm.

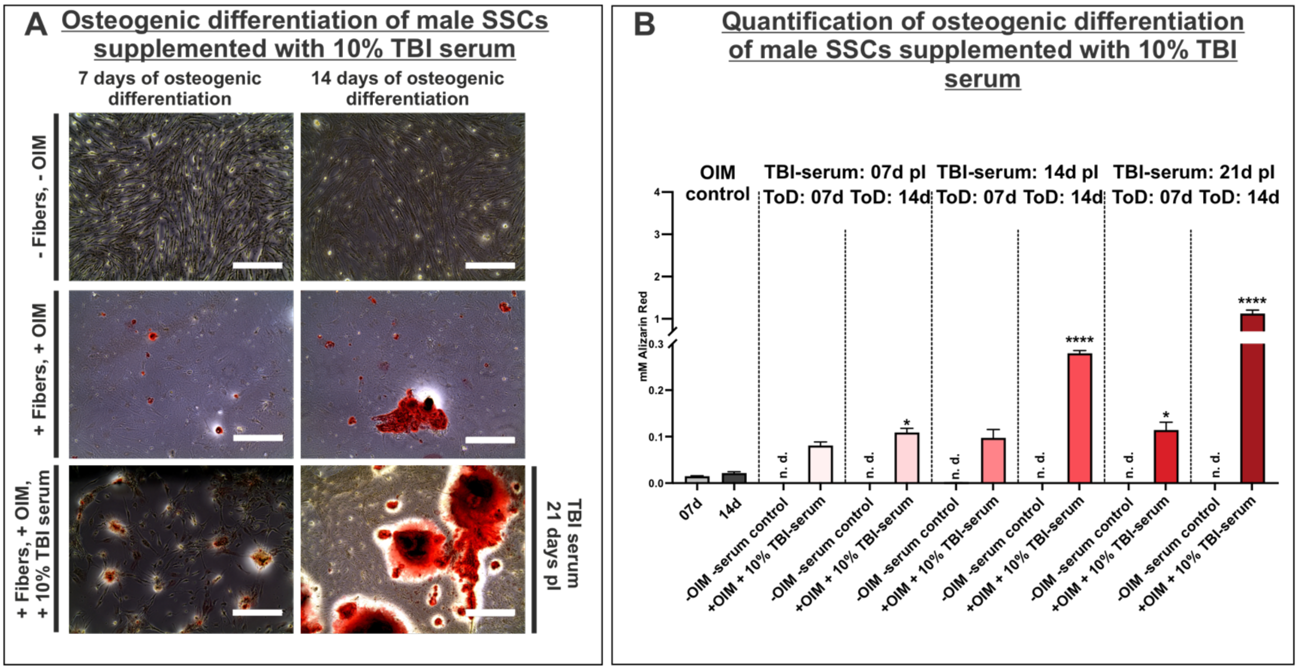

Figure S 3 **SSCs derived from female donor 6 revealed enhanced osteogenic differentiation potential in response to TBI sera.** (A,B) Osteogenic differentiation of SSCs derived from female donor 6 differentiated for 14 days with the supplementation of TBI serum 14 and 21 days pI led to calcified areas indicating a high osteogenic differentiation potential in comparison to OIM control (depicted in red by Alizarin Red staining). Enhanced osteogenic differentiation potential was also detected when cells were cultivated in TBI serum 7 days pI (differentiated for 14 days) and 14 days pI incubated for 7 days.Data are shown as mean ± SD. All samples were statistically assessed with an ordinary one-way ANOVA relative to the OIM control group, **** $p < 0.0001$ was considered significant. Scale bars in image A represent 400 µm.

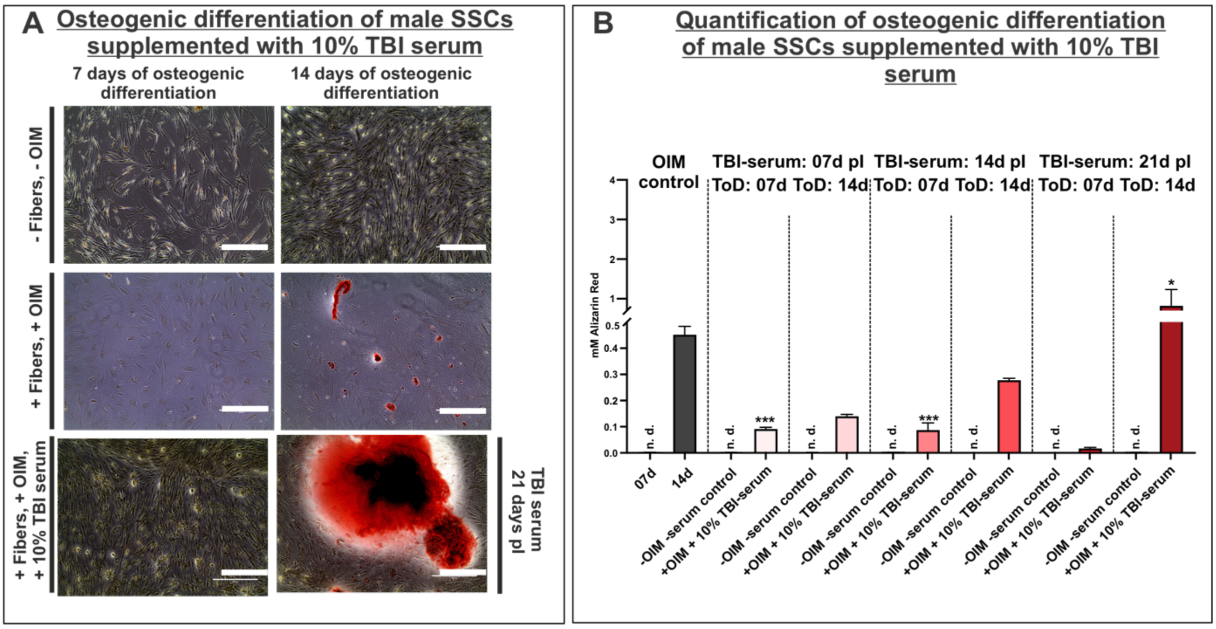

Figure S 4 **SSCs derived from female donor 7 revealed enhanced osteogenic differentiation potential in response to TBI sera.** (A,B) Osteogenic differentiation of SSCs derived from female donor 7 differentiated for 14 days with the supplementation of TBI serum 21 days pI led to calcified areas indicating a high osteogenic differentiation potential in comparison to OIM control (depicted in red by Alizarin Red staining). Enhanced osteogenic differentiation potential was also detected when cells were cultivated in TBI serum 7 and 14 days pI incubated for 7 days. Data are shown as mean ± SD. All samples were statistically assessed with an ordinary one-way ANOVA relative to the OIM control group, **** $p < 0.0001$ was considered significant. Scale bars in image A represent 400 µm

Supplementary Table 1: Overview of medications administered to patient TBI M1 at 7, 14 and 21 days post-injury. The table lists the active substance, total dose, start of administration (S.o.A) and end of administration (E.o.A)

Medication administered up to blood collection at 7 days post-injury				Medication administered up to blood collection at 14 days post-injury				Medication administered up to blood collection at 21 days post-injury			
**Active substance**	**Total dose**	**S.o.A**	**E.o.A**	**Active Substance**	**Total dose**	**S.o.A**	**E.o.A**	**Active substance**	**Total Dose**	**S.o.A**	**E.o.A**
Cefotaxime	23824 mg	d3	d6	Ciprofloxacin	8800 mg	d8	d14	Clindamycin	15.8 mg	15	21
Ciprofloxacin	1600 mg	d6	d7	Metamizole	2 g	d9	d9	Fentanyl	0.86 mg	19	22
Clonidine	1.5 mg	d6	d7	Paracetamol	1000 mg	d8	d8	Metoclopramide	130 mg	15	21
Furosemide	55 mg	d2	d7	Clonidine	0.75 mg	d6	d7	Morphine	3 mg		
Metamizole	1 g	d3	d3	Midazolam	300 mg	d8	d14	Clonidine	4.5 mg	21	21
Midazolam	300 mg	d2	d7	Propofol	23,1 g	d11	d14	Propofol	13.73 g	15	21
Norepinephrine	27 mg	d0	d7	Sufentanil	1.87 mg	d8	d14	Sufentanil	1.87 mg	11	14
Norepinephrine	27 mg	d2	d7	Norepinephrine	30 mg	d8	d14	Norepinephrine	20 mg	15	21
Paracetamol	1000 mg	d7	d7								
Propofol	11.65 g	d0	d2								
Propofol	11.65 g	d6	d7								
Rocuronium bromide	50 mg	d7	d7								
Sufentanil	1 mg	d0	d2								
Sufentanil	1 mg	d7	d7								

Supplementary Table 2: Overview of medications administered to patient TBI M2 at 7, 14 and 21 days post-injury. The table lists the active substance, total dose, start of administration (S.o.A) and end of administration (E.o.A)

Medication administered up to blood collection at 7 days post-injury			
**Active substance**	**Total dose**	**S.o.A**	**E.o.A**
Ampicillin/Sulbactam	84 g	d1	d1
Furosemide	10mg	d0	d0
Hydrocortisone	100 mg	d0	d2
Desmopressin	0.1 mg	d0	d0
Norepinephrine	1 mg	d0	d0
Paracetamol	1000 mg	d0	d0
Paracetamol	1000 mg	d4	d4
Propofol	15 g	d0	d3
Sufentanil	6 mg	d0	d7

Medication administered up to blood collection at 14 days post-injury			
**Active Substance**	**Total dose**	**S.o.A**	**E.o.A**
Ampicillin/Sulbactam	84 g	d8	d8
Ferric carboxymaltose	1000 mg	d8	d8
Haloperidol	3 mg	d8	d8
Metamizole	35 g	d8	d14
Desmopressin	0.4 mg	d8	d8
Pantoprazole	60 mg	d12	d14
Pipamperone	240 mg	d12	d14
Cyanocobalamin	400 mg	d8	d9

Medication administered up to blood collection at 21 days post-injury			
**Active substance**	**Total Dose**	**S.o.A**	**E.o.A**
Promethazine	50 mg	d21	d21
Bisacodyl	10 mg	d16	d16
Cefuroxime	18 g	d15	d19
Clonidine	0.75 mg	d15	d15
Diazepam	10 mg	d20	d21
Piritramide	3.75 mg	d17	d17
Ferric carboxymaltose	1000 mg	d15	d15
Haloperidol	4 mg	d21	d21
Pethidine	100 mg	d19	d19
Metamizole	100 mg	d19	d19
Pantoprazole	3 g	d15	d19
Quetiapine	160 mg	d15	d21

Supplementary Table 3: Overview of medications administered to patient TBI M3 at 7, 14 and 21 days post-injury. The table lists the active substance, total dose, start of administration (S.o.A) and end of administration (E.o.A)

Medication administered up to blood collection at 7 days post-injury				Medication administered up to blood collection at 14 days post-injury				Medication administered up to blood collection at 21 days post-injury			
**Active substance**	**Total dose**	**S.o.A**	**E.o.A**	**Active Substance**	**Total dose**	**S.o.A**	**E.o.A**	**Active substance**	**Total Dose**	**S.o.A**	**E.o.A**
Metamizole	1000 mg	d6	d6	Cefotaxime	12 g	d7	d12	Clonidine	1,5 mg	17	17
Fludrocortisone	0.2 mg	d6	d6	Clonidine	1.5 mg	d8	d12	Metoclopramide	180 mg	13	18
Cefotaxime	6 g	d4	d6	Ketamine	80 mg	d8	d8	Metamizole	30 g	13	13
Clindamycin	600 mg	d0	d0	Metoclopramide	150 mg	d8	d13	Risperidone	12 mg	13	18
Clonidine	75 µg	d6	d7	Metamizole	15 g	d10	d12				
Ferric carboxymaltose	1000 mg	d4	d4	Risperidone	8 mg	d10	d12				
Furosemide	10 mg	d6	d6	Propofol	4.5 g	d7	d9				
Hydrocortisone	40 mg	d2	d5								
Ketamine	1400 mg	d2	d7								
Cisatracurium besilate	10 mg	d0	d0								
Paracetamol	1000 mg	d5	d5								
Propofol	7 g	d1	d6								
Sufentanil	7 mg	d1	d6								

Supplementary Table 4: Overview of medications administered to patient TBI M4 at 7, 14 and 21 days post-injury. The table lists the active substance, total dose, start of administration (S.o.A) and end of administration (E.o.A)

Medication administered up to blood collection at 7 days post-injury

Active substance	Total dose	S.o.A	E.o.A
Paracetamol	2000 mg	d5	d6
Cefotaxime	4 g	d6	d7
Propofol	29.2 g	d0	d6
Sufentanil	8 mg	d1	d7
Tranexamic acid	250 mg	d1	d1
Dobutamine	812.5 mg	d4	d7
Norepinephrine	80 mg	d1	d7

Medication administered up to blood collection at 14 days post-injury

Active Substance	Total dose	S.o.A	E.o.A
Paracetamol	1000 mg	d11	d11
Levothyroxine	250 µg	d9	d13
Metamizole	2g	d11	d12
Cefotaxime	6 g	d8	d13
Dexmedetomidine	2800 µg	d8	d14
Flucloxacillin	1750 mg	d8	d14
Metamizole	10 g	d13	d14
Propofol	1.57 g	d8	d14
Dobutamine	83 mg	d8	d8
Norepinephrine	62 mg	d8	d14

Medication administered up to blood collection at 21 days post-injury

Active substance	Total Dose	S.o.A	E.o.A
Levothyroxine	750 µg	16	21
Torasemide	50 mg	15	21
Piritramide	29.75 mg	15	21
Parecoxib	240 mg	15	21
Dexmedetomidine	600 µg	15	16
Flucloxacillin	1750 mg	15	21
Metamizole	35 g	15	21
Norepinephrine	3.19 mg	15	20

Supplementary Table 5: Overview of medications administered to patient TBI F1 at 7, 14 and 21 days post-injury. The table lists the active substance, total dose, start of administration (S.o.A) and end of administration (E.o.A)

Medication administered up to blood collection at 7 days post-injury

Active substance	Total dose	S.o.A	E.o.A
Piritramide	3.75 mg	d7	d7
Urapidil	12.5 mg	d7	d7
Pantoprazole	45.11 mg	d7	d7

Medication administered up to blood collection at 14 days post-injury

Active Substance	Total dose	S.o.A	E.o.A
Fosfomycin	5.361 g	d10	d10
Tilidine	411.6 mg	d9	d12
Metamizole	24 g	d8	d13
Levetiracetam	2000 mg	d13	d13

Medication administered up to blood collection at 21 days post-injury

Active substance	Total Dose	S.o.A	E.o.A
Ibuprofen	10800 mg	13.07.22	18.07.22
Metamizole	28 g	11.07.22	18.07.22
Levetiracetam	14000 mg	11.07.22	18.07.22

Supplementary Table 6: Overview of medications administered to patient TBI F2 at 7, 14 and 21 days post-injury. The table lists the active substance, total dose, start of administration (S.o.A) and end of administration (E.o.A)

Medication administered up to blood collection at 7 days post-injury				Medication administered up to blood collection at 14 days post-injury				Medication administered up to blood collection at 21 days post-injury			
**Active substance**	**Total dose**	**S.o.A**	**E.o.A**	**Active Substance**	**Total dose**	**S.o.A**	**E.o.A**	**Active substance**	**Total Dose**	**S.o.A**	**E.o.A**
Bisoprolol	10 mg	d6	d7	Amlodipine	30 mg	d9	d14	Amlodipine	35 mg	d15	d21
Midazolam	5 mg	d3	d3	Bisoprolol	35 mg	d8	d14	Bisoprolol	35 mg	d15	d21
Clobazam	60 mg	d2	d7	Hydrochlorothiazide	87.5 mg	d8	d14	Hydrochlorothiazide	87.5 mg	d15	d21
Furosemide	10 mg	d3	d3	Levetiracetam	16000 mg	d10	d14	Levetiracetam	24500 mg	d15	d21
Hydrochlorothiazide	12.5 mg	d7	d7	Ramipril	55 mg	d8	d14	Pantoprazole	480 mg	d17	d21
Ramipril	10 mg	d6	d7	Torasemide	15 mg	d13	d14	Ramipril	70 mg	d15	d21
Clonidine	4.3 mg	d2	d5	Cefotaxime	14 g	d8	d14	Torasemide	35 mg	d15	d21
Propofol	4.14 g	d2	d3	Clonidine	6,5 mg	d9	d13	Cefotaxime	14 g	d15	d21
Sufentanil	0.63 mg	d2	d3	Urapidil	785.5 mg	d8	d9	Vancomycin	3500 mg	d15	d21
Urapidil	553 mg	d6	d7	Vancomycin	3500 mg	d8	d14				
Vancomycin	500 mg	d7	d7								
Norepinephrine	18.1 mg	d2	d7								

Supplementary Table 7: Overview of medications administered to patient TBI F3 at 7, 14 and 21 days post-injury. The table lists the active substance, total dose, start of administration (S.o.A) and end of administration (E.o.A)

Medication administered up to blood collection at 7 days post-injury				Medication administered up to blood collection at 14 days post-injury				Medication administered up to blood collection at 21 days post-injury			
**Active substance**	**Total dose**	**S.o.A**	**E.o.A**	**Active Substance**	**Total dose**	**S.o.A**	**E.o.A**	**Active substance**	**Total Dose**	**S.o.A**	**E.o.A**
Amlodipine	25 mg	d4	d7	Amlodipine	65 mg	d8	d14	Folic acid	30 mg	d15	d21
Bisoprolol	5 mg	d4	d6	Bisoprolol	17.5 mg	d8	d14	Bisoprolol	17.5 mg	d15	d21
Candesartan	128 mg	d4	d7	Candesartan	224 mg	d8	d14	Amlodipine	65 mg	d15	d21
Folic acid	5 mg	d6	d6	Folic acid	30 mg	d8	d13	Candesartan	224 mg	d15	d21
Pantoprazole	280 mg	d1	d7	Pantoprazole	280 mg	d8	d14	Paracetamol + Codeine phosphate	7000 mg/630 mg	d15	d21
Piritramide	30 mg	d1	d4	Piritramide	9 mg	d13	d13	Risperidone	6 mg	d19	d21
Nifedipine	225 mg	d2	d5	Paracetamol	2000 mg	d10	d11	Pipamperone	23.8 mg	d18	d18
Paracetamol	2000 mg	d5	d6	Cefotaxime	8 g	d8	d11				
Cefotaxime	6 g	d5	d7	Metamizole	15 g	d8	d11				
Clonidine	0.75 mg	d7	d7	Urapidil	160 mg	d8	d11				
Metamizole	35 g	d1	d7								
Propofol	3,9 g	d5	d7								
Sufentanil	1 mg	d5	d6								
Urapidil	716 mg	d3	d5								
Norepinephrine	3.25 mg	d4	d5								

Supplementary Table 8: Overview of medications administered to patient TBI F4 at 7, 14 and 21 days post-injury. The table lists the active substance, total dose, start of administration (S.o.A) and end of administration (E.o.A)

Medication administered up to blood collection at 7 days post-injury			
**Active substance**	**Total dose**	**S.o.A**	**E.o.A**
Amiodarone	4500 mg	d4	d7
Argipressin	665 mg	d3	d7
Cefotaxime	8 g	d1	d4
Clonidine	3.75 mg	d0	d5
Hydrocortisone	50 mg	d3	d7
Isoflurane	1 Vol.-%	d4	d7
Metoprolol	5 mg	d4	d4
Norepinephrine	19.2 mg	d0	d7
Pantoprazole	280 mg	d0	d7
Paracetamol	1000 mg	d4	d4
Propofol	15.4 g	d0	d7
Sufentanil	7 mg	d0	d7
Piperacillin/Tazobactam	54 g	d4	d7
Torasemide	15 mg	d5	d7
Lidocaine	100 mg	d4	d4

Medication administered up to blood collection at 14 days post-injury			
**Active Substance**	**Total dose**	**S.o.A**	**E.o.A**
Argipressin	399 mg	d10	d12
Clonidine	3 mg	d11	d14
Hydrocortisone	40 mg	d8	d11
Isoflurane	1 Vol.-%	d8	d11
Metoprolol	332.5 mg	d8	d14
Norepinephrine	16.3 mg	d8	d14
Metamizole	2 g	d9	d10
Pantoprazole	280 mg	d8	d14
Sufentanil	6 mg	d8	d13
Piperacillin/Tazobactam	67.5 g	d8	d12
Torasemide	35 mg	d8	d14

Medication administered up to blood collection at 21 days post-injury			
**Active substance**	**Total Dose**	**S.o.A**	**E.o.A**
Clonidine	4.5 mg	d16	d21
Furosemide	10 mg	d19	d19
Metamizole	10 g	d19	d20
Metoprolol	332.5 mg	d16	d21
Norepinephrine	14.4 mg	d16	d21
Pantoprazole	280 mg	d16	d21
